# Supplementary material for: Aberrant Expression of Intracellular let-7e, miR-146a, and miR-155 Correlates with Severity of Depression in Patients with Major Depressive Disorder and Is Ameliorated after Antidepressant Treatment
Source: Cells. 2019 Jun 27;8(7):647. doi: 10.3390/cells8070647 (PMC6678487; doi:10.3390/cells8070647)
Supplement: Supplementary file 1 [file cells-08-00647-s001.pdf]

Supplement Table 1. Sequences of mature microRNAs

Assay ID	Assay Name	Mature microRNA Sequence (5′→3′)
002406	hsa-let-7e	UGAGGUAGGAGGUUGUAUAGUU
000397	hsa-miR-21-5p	UGAGGUAGUAGGUUGUAUGGUU
002278	hsa-miR-145	GUCCAGUUUUCCCAGGAAUCCCU
000468	hsa-miR-146a	UGAGAACUGAAUUCCAUGGGUU
002623	hsa-miR-155	UUAAUGCUAAUCGUGAUAGGGGU
002295	hsa-miR-223	UGUCAGUUUGUCAAAUACCCCA
001973		U6

Supplement Table 2. Comparison of mRNA expression levels (-ΔCt) of negative regulators in MDD patients before and after two different antidepressant treatments

	SSRIs (n = 21)		*p*-value	SNRIs (n = 32)		*p*-value
	Pre-treatment	Post-treatment		Pre-treatment	Post-treatment	
let-7e	-4.53 ± 2.28	-3.39 ± 1.71	$p = 0.008$**	-4.13 ± 1.37	-3.91 ± 1.31	$p = 0.417$
miR-21-5p	-6.75 ± 2.74	-6.08 ± 2.41	$p = 0.277$	-5.52 ± 1.67	-5.38 ± 1.60	$p = 0.665$
miR-223	2.19 ± 2.30	3.22 ± 1.78	$p = 0.014$*	3.34 ± 1.26	3.46 ± 1.55	$p = 0.497$
miR-145	-5.91 ± 1.54	-5.21 ± 1.47	$p = 0.010$*	-5.89 ± 1.45	-5.93 ± 1.59	$p = 0.910$
miR-146a	-2.88 ± 2.99	-2.32 ± 2.81	$p = 0.181$	-1.33 ± 1.35	-1.17 ± 1.48	$p = 0.505$
miR-155	-4.08 ± 2.73	-3.07 ± 1.97	$p = 0.006$**	-2.93 ± 1.03	-2.81 ± 1.09	$p = 0.570$

Mann-Whitney Test was used to compare differences between pre- and post-treatment in SSRI group.
A paired sample t-test was used to compare differences between pre- and post-treatment in SNRI group.
GAPDH was used as a housekeeping gene to assess the relative abundance of mRNA; **p*-value < 0.05, ***p*-value < 0.01
SSRIs, selective serotonin reuptake inhibitors; SNRIs, Serotonin–norepinephrine reuptake inhibitors
